# scOTM: A Deep Learning Framework for Predicting Single-Cell Perturbation Responses with Large Language Models

**DOI:** 10.3390/bioengineering12080884

**Published:** 2025-08-20

**Authors:** Yuchen Wang, Tianchi Lu, Xingjian Chen, Zhongyu Yao, Ka-Chun Wong

**Affiliations:** 1 Department of Computer Science, City University of Hong Kong, Kowloon Tong, Hong Kong SAR 999077, China; 2 Cutaneous Biology Research Center, Massachusetts General Hospital, Harvard Medical School, Boston, MA 02148, USA

**Keywords:** single-cell perturbation prediction, deep learning, optimal transport, large language model

## Abstract

Modeling drug-induced transcriptional responses at the single-cell level is essential for advancing human healthcare, particularly in understanding disease mechanisms, assessing therapeutic efficacy, and anticipating adverse effects. However, existing approaches often impose a rigid constraint by enforcing pointwise alignment of latent representations to a standard normal prior, which limits expressiveness and results in biologically uninformative embeddings, especially in complex biological systems. Additionally, many methods inadequately address the challenges of unpaired data, typically relying on naive averaging strategies that ignore cell-type specificity and intercellular heterogeneity. To overcome these limitations, we propose scOTM, a deep learning framework designed to predict single-cell perturbation responses from unpaired data, focusing on generalization to unseen cell types. scOTM integrates prior biological knowledge of perturbations and cellular states, derived from large language models specialized for molecular and single-cell corpora. These informative representations are incorporated into a variational autoencoder with maximum mean discrepancy regularization, allowing flexible modeling of transcriptional shifts without imposing a strict constraint of alignment to a standard normal prior. scOTM further employs optimal transport to establish an efficient and interpretable mapping between control and perturbed distributions, effectively capturing the transcriptional shifts underlying response variation. Extensive experiments demonstrate that scOTM outperforms existing methods in predicting whole-transcriptome responses and identifying top differentially expressed genes. Furthermore, scOTM exhibits superior robustness in data-limited settings and strong generalization capabilities across cell types.

## 1. Introduction

Cells are the fundamental units of life, and understanding how they respond to external perturbations, such as drugs, cytokines, or genetic modifications, is critical for advancing systems pharmacology and human healthcare [1]. Current human cell atlases provide extensive snapshots of cells in static states, but remain severely limited in capturing cellular responses to perturbations such as drug treatments. Experimentally acquiring such dynamic response data at scale is constrained by high cost, labor intensity, and substantial time requirements. These challenges call for the development of robust computational frameworks capable of modeling complex transcriptional responses to drug perturbations and generalizing to unseen cell types. Recent advances in artificial intelligence (AI) have enabled the development of virtual cell models, computational frameworks designed to simulate cellular responses using large-scale single-cell data [2]. However, existing approaches often face significant challenges when learning from sparse, noisy, unpaired perturbation scRNA-seq datasets [3]. Therefore, developing robust computational methods to address the challenges of virtual cell simulation still remains a pressing research priority.

IFN-β is a clinically approved immunomodulatory cytokine, widely used in the treatment of autoimmune diseases such as multiple sclerosis [4]. Upon administration, IFN-β activates the JAK-STAT signaling cascade and induces a broad transcriptional program involving interferon-stimulated genes (ISGs), modulating antiviral defense, inflammation, and cell proliferation [5,6]. While the therapeutic mechanisms of IFN-β have been extensively studied in bulk assays, the emergence of single-cell RNA sequencing (scRNA-seq) now enables high-resolution dissection of cell-type-specific transcriptional responses to IFN-β [7]. This is particularly important given the substantial heterogeneity in IFN-β sensitivity observed across immune cell types and disease contexts [8]. Accurate prediction of IFN-β-induced gene expression changes at single-cell resolution holds great potential for elucidating the cellular basis of drug action, identifying responsive or resistant subpopulations, and guiding precision immunotherapy [9]. In addition to cytokine-based perturbations, IFN-β, we also investigated responses to belinostat, a clinically approved Histone Deacetylase (HDAC) inhibitor used in the treatment of T-cell lymphomas [10]. Belinostat alters gene expression by modulating chromatin accessibility, and its effects are known to vary across immune cell types. This makes it a suitable candidate for evaluating the ability of computational models to capture heterogeneous transcriptional responses at single-cell resolution.

**Figure 1:**
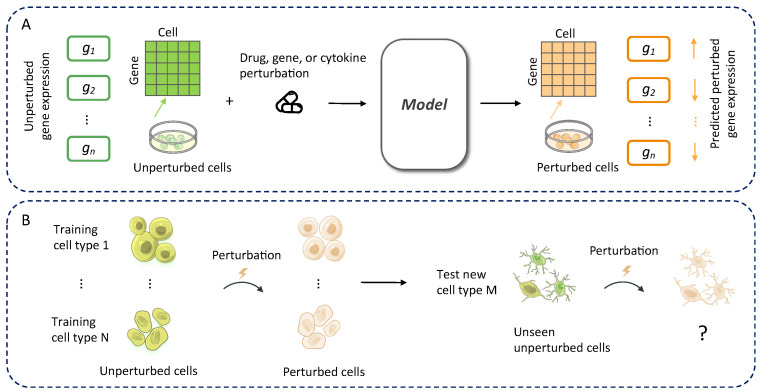
(**A**) General process of computational modeling for perturbation prediction. The model aims to predict the cellular transcriptional response (orange) to drug-induced perturbations across different cell types from the unperturbed status (green). (**B**) Model generalization across cell types. The key challenge in computational modeling for perturbation prediction is generalizing from observed cell types to unseen samples from a new cell type.

Modeling drug-induced transcriptional changes from unpaired control and stimulated single-cell datasets presents several key challenges. First, discrepancies in cell type composition between conditions introduce distributional shifts that confound direct comparisons, making it difficult to disentangle true drug responses from population-level differences [11]. Second, the absence of one-to-one correspondence between cells under control and stimulated states limits the ability to learn direct perturbation mappings at the individual cell level [12]. Third, biological and technical variability, such as batch effects, donor heterogeneity, and cell cycle stages, can further obscure drug-specific transcriptional signals when no paired baseline exists for each cell [13]. These factors collectively hinder the development of accurate and generalizable models, as the learning process must infer both cell identity alignment and perturbation effects simultaneously from globally mismatched populations [14]. Figure 1 illustrates the general process of computational modeling for perturbation prediction and highlights the key challenge that is generalizing from observed cell types to unseen samples of a new cell type.

### Related Works

In recent years, several studies have explored deep learning methods to predict perturbation responses in single-cell genomics [15]. Representative methods using the variational autoencoder (VAE) structure include scGen [12], trVAE [16], and scVIDR [17]. Both scGen and scVIDR utilize variational autoencoders to learn latent representations of cellular states and simulate perturbation effects via vector operations in the latent space [12]. scVIDR extends this approach by incorporating a linear regression model to predict perturbation vectors for previously unseen cells [17]. Nonetheless, scGen overlooks the pronounced heterogeneity in perturbation responses across distinct cell types, relying instead on the simplistic averaging of perturbation vectors within each type. Although scVIDR considers inter-cell type variation, it overlooks intra-cell type diversity, and its dependence on limited training data for the regression component can hinder its ability to accurately capture complex perturbation dynamics. trVAE addresses the challenge of unpaired data by leveraging a conditional VAE framework [18], enhanced with a maximum mean discrepancy objective to enable style transfer between the control and perturbed conditions [16,19]. However, its reliance on randomly pairing samples from the control and perturbation groups during training can disrupt the preservation of intrinsic biological structure, potentially resulting in suboptimal alignment and a reduced capacity to model coherent perturbation trajectories.

Originally developed in the fields of mathematics and economics [20], optimal transport offers a powerful mathematical tool for aligning probability distributions in an efficient manner. CellOT [21] adapts this concept to the single-cell domain by employing input convex neural networks [22] to learn direct mappings from control to perturbed cellular states. Despite its effectiveness on training data, CellOT exhibits poor generalization when confronted with data distributions that differ from those encountered during training [23]. scPRAM [23] integrates a standard variational autoencoder to learn latent representations and employs optimal transport to estimate perturbation dynamics. However, its dependence on pointwise Kullback–Leibler divergence and rigid enforcement of prior distribution alignment can constrain the flexibility of the latent space, often resulting in subpar representations when modeling the intricacies of complex biological systems.

Recent breakthroughs in single-cell large language models (LLMs) have opened new avenues for biological discovery [24]. Cutting-edge models such as scGPT [25], trained on tens of millions of single-cell transcriptomes, demonstrate remarkable capabilities in downstream tasks. However, directly applying these large-scale models to predict perturbation response, whether through training or fine-tuning, still imposes substantial computational demands [24]. To address these challenges, we propose a computational framework with optimal transport (OT) and maximum mean discrepancy (MMD) regularization (scOTM) that predicts single-cell perturbation responses with high fidelity and strong generalization across unseen conditions. By combining LLM-encoded embeddings and leveraging MMD-based latent regularization with OT-driven distribution mapping, scOTM offers a flexible yet principled solution to model transcriptional shifts without requiring paired data.

## 2. Materials and Methods

### 2.1. Datasets and Preprocessing

We use publicly available datasets for evaluation, including the human peripheral blood mononuclear cell (PBMC) dataset from Kang et al. [26] and a publicly available dataset from the Open Problems—Single-Cell Perturbations competition on Kaggle [27], which consists of human PBMCs that were subjected to perturbations by a panel of compounds. We focused on the subset of cells treated with belinostat and their corresponding DMSO controls, as belinostat is an FDA-approved HDAC inhibitor for T-cell lymphoma, inducing robust and interpretable transcriptional perturbations in single-cell data, forming a paired dataset with 8562 control cells and 8101 perturbed cells. The original data can be accessed from the Gene Expression Omnibus (GEO) database with identifier GSE96583 [26]. The Kaggle datasets can be downloaded from the Kaggle competition webpage (accessed on 17 August 2025) [27].

**Figure 2:**
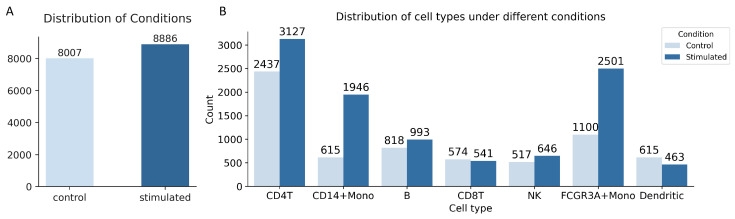
The distribution of the PBMC dataset. (**A**) The distribution of control and perturbed counts. (**B**) The distribution of control and perturbed counts according to cell types.

For data preprocessing, to ensure fairness and consistency in the comparisons, we followed the same preprocessing strategy as adopted in the scGen [12] framework. Specifically, the dataset was first filtered to retain cells with at least 500 expressed genes and genes expressed in at least 5 cells. Next, library size normalization was performed by scaling total counts per cell to a target value, followed by log-transformation of the normalized counts, following the standard practice in scRNA-seq analysis. Finally, the top 6998 highly variable genes were selected for downstream modeling. The distribution of the Kang PBMC dataset in Figure 2 provides a quantitative overview of cell composition and count across conditions, where panel A presents the total number of cells under each condition, with 8007 cells in the control group and 8886 in the stimulated group, and panel B shows the overall counts of each cell type across all samples. The PBMC dataset comprises seven distinct cell types, enabling the design of seven independent sub-experiments. We initially utilized the cell type annotations to define the training and testing sets, which are subsequently excluded during the training process and inference process.

For each sub-experiment, we hold out all data from a specific cell type under the stimulated condition to serve as the test set, while the remaining data constitute the training set. The training set includes cells under both the control condition (train control) and the stimulated condition (train stimulated), excluding the held-out cell type. Similarly, the testing set comprises two parts: cells of the held-out type under the control condition (test control) and their corresponding real stimulated profiles (test stimulated). During training, all training data are used to optimize the model. At test time, test control cells are fed into scOTM to generate predicted test stimulated responses, which are then compared to the ground-truth test stimulated cells for evaluation.

To guarantee a fair comparison with alternative approaches, we maintain the same training set for all methods and evaluate the performance of each model on the same testing set.

### 2.2. Model Framework

**Figure 3:**
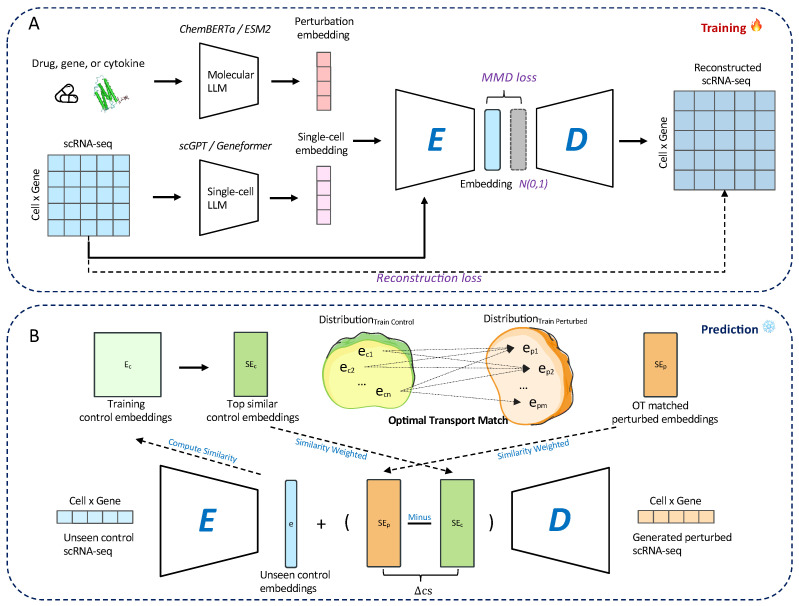
Overview of scOTM framework. (**A**) Training pipeline. scOTM encodes both control and perturbed single-cell transcriptomic profiles into a shared latent embedding space, where MMD loss is applied to align the aggregated latent distribution with the standard normal prior. The drug, gene, or cytokine is first encoded using a molecular LLM such as ChemBERTa or ESM2 to obtain a perturbation embedding, while the scRNA-seq profile is encoded using a single-cell LLM (e.g., scGPT or Geneformer) to generate a single-cell embedding. scRNA-seq profile and embeddings are jointly passed through an encoder *E*, which projects them into a latent space regularized by MMD loss to approximate a standard Gaussian prior N(0,1). The decoder *D* reconstructs the scRNA-seq expression matrix from the latent embedding. (**B**) Prediction step. At inference time, for each unseen control cell, scOTM encodes the input into the latent space and retrieves its most similar counterparts from the training control embeddings. Then, optimal transport is employed to estimate the corresponding perturbed embedding by aligning the control and perturbed distributions in the latent space. Finally, the predicted perturbed response is obtained by decoding the aligned embedding using the scOTM decoder.

We introduce scOTM, a generative model for predicting single-cell perturbation responses from unpaired data (see Figure 3). The scOTM framework integrates a maximum mean discrepancy variational autoencoder (MMD-VAE) and optimal transport to model perturbation responses from single-cell transcriptomics (see Figure 3). In this paper, *P* represents perturbation and $D\in R^{n\times d}$ denotes the single-cell expression matrix, where *n* is the number of cells, *d* is the number of genes, $D_{\mathrm{ctr}}\in R^{n_{c}\times d}$ represents the control group (unperturbed profiles), and $D_{\mathrm{ptb}}\in R^{n_{p}\times d}$ corresponds to the perturbed (also called stimulated) profiles.

To incorporate prior biological insights from both single-cell profiles and perturbation knowledge, we integrate embeddings extracted from LLMs into the model input. Specifically, we utilize two types of LLMs: (1) an $LLM_{sc}$ model, such as scGPT [25] pretrained on millions of gene expression data, is used to extract embeddings of single-cell profiles; and (2) an $LLM_{p}$ model, such as ChemBERTa [28] and ESM2 [29], pretrained on molecular structures or protein sequences, is used to generate embeddings for perturbations. These embeddings are then element-wise added to the corresponding gene expression vector D before being passed into the encoder. The perturbation embeddings enable the model to explicitly capture the semantic shift between control and stimulated conditions, while the single-cell profile embeddings provide a biologically informed initialization that enhances the representation of cell identity.

The MMD-VAE then learns the underlying data distribution and projects the high-dimensional input into a lower-dimensional latent space. It consists of two core components: an *encoder* network that transforms the input $D+D_{\mathrm{embed}}+P_{\mathrm{embed}}$ into a latent representation $z\in R^{k}$, and a *decoder* network that reconstructs the original expression $\hat{D}$ from this compressed code.

(1) $$D_{\mathrm{embed}}=LLM_{sc}\left(D\right)$$

(2) $$P_{\mathrm{embed}}=LLM_{p}\left(P\right)$$

(3) $$z=Encoder(D+D_{\mathrm{embed}}+P_{\mathrm{embed}})$$

(4) $$\hat{D}=Decoder(z)$$

The latent variable z is sampled using the reparameterization trick from a Gaussian distribution parameterized by μ and σ:

(5) z=μ+ϵ⊙σ,ϵ∼N(0,I)

where ⊙ denotes element-wise multiplication.

To align the aggregated posterior distribution of the latent variable z with the standard normal prior, we replace the conventional Kullback–Leibler (KL) divergence with the MMD loss. Unlike KL divergence, which requires an explicit probability density function, MMD is a non-parametric, kernel-based statistical measure that compares the empirical distributions of samples drawn from z and the standard Gaussian prior. The discrepancy is evaluated using a Gaussian kernel and penalizes differences between distributions in the associated reproducing kernel Hilbert space.

The overall objective function of scOTM combines two loss components:

(6) $$L_{total}=L_{recon}\left(D,D^{\prime }\right)+\alpha \cdot L_{\mathrm{MMD}}\left(z,z_{\mathrm{prior}}\right)$$

with the individual loss terms defined as:

(7) $$z_{\mathrm{prior}}∼N\left(0,I\right)$$

(8) $$L_{recon}\left(D,D^{\prime }\right)={∥D-\hat{D}∥}^{2}$$

(9) $$L_{\mathrm{MMD}}={\mathrm{MMD}}^{2}\left(q(z)\;∥\;N(0,I)\right)$$

where α is a weighting hyperparameter that balances the reconstruction and regularization terms. For the selection strategy for α, we first ensured that the magnitudes of reconstruction and regularization MMD losses were within a comparable range, setting their ratio between [0.1, 10]. We then performed a grid search within this range to determine the optimal value of the hyperparameter α for the MMD regularization loss.

The ${\mathrm{MMD}}^{2}$ is computed using a Gaussian kernel function k(·,·) as follows:

(10) $$\begin{array}{cc} L_{\mathrm{MMD}}= & E_{z,z^{\prime }}\left[k\left(z,z^{\prime }\right)\right]+E_{z_{p},z_{p}^{\prime }}\left[k\left(z_{p},z_{p}^{\prime }\right)\right]\\ & -2E_{z,z_{p}}\left[k\left(z,z_{p}\right)\right] \end{array}$$

where $z,z^{\prime }∼q\left(z\right)$, and $z_{p},z_{p}^{\prime }∼N\left(0,I\right)$. In our implementation, we use a Gaussian radial basis function (RBF) kernel defined by:

(11) $$k\left(x,y\right)=\exp\left(-\frac{1}{2\sigma^{2}}{∥x-y∥}^{2}\right)$$

where σ is the kernel bandwidth controlling the smoothness of the kernel and the sensitivity of the MMD loss to differences between the latent representations and the normal distribution. For the selection of bandwidth, after evaluating both adaptive bandwidths by median heuristic and grid search, σ equal to 1 or 2 yielded the most competitive performance. This is attributable to both inter-cell-type heterogeneity and intra-cell-type variability.

### 2.3. Optimal Transport-Based Alignment Between Cell States

To model the correspondence between control and perturbed (also called stimulated) cell states in training sets, we first encode gene expression profiles into a shared latent space using an encoder network. The latent representations are denoted as

(12) $$Z_{\mathrm{ctrl}}={\left\{z_{i}^{\mathrm{ctrl}}\right\}}_{i=1}^{n},\;Z_{\mathrm{stim}}={\left\{z_{j}^{\mathrm{stim}}\right\}}_{j=1}^{m}$$

where each $z\in R^{d}$ is a low-dimensional embedding of a single cell in either the control or stimulated condition.

We formulate the alignment between the two distributions in latent space as an optimal transport problem. Specifically, we define a cost matrix $C\in R^{n\times m}$, where each entry represents the squared Euclidean distance between control and stimulated cells:

(13) $$\begin{array}{c} C_{ij}={∥z_{i}^{\mathrm{ctrl}}-z_{j}^{\mathrm{stim}}∥}^{2} \end{array}$$

The Earth Mover’s Distance formulation is then solved to obtain the optimal transport plan [30]:

(14) $$\begin{array}{c} \min_{\gamma \in \Pi (\mu ,\nu )}\sum_{i=1}^{n}\sum_{j=1}^{m}\gamma_{ij}C_{ij} \end{array}$$

subject to the marginal constraints $\gamma 1_{m}=\mu$ and $\gamma^{⊤}1_{n}=\nu$, where $\mu \in R^{n}$ and $\nu \in R^{m}$ are uniform distributions over the control and stimulated populations, respectively.

The resulting optimal coupling $\gamma \in R_{+}^{n\times m}$ encodes a soft alignment between control and stimulated cells. Each entry $\gamma_{ij}$ represents the amount of probability mass transported from control cell *i* to stimulated cell *j*, forming a probabilistic correspondence used for new sample prediction.

### 2.4. Predicting the Perturbed State

Given a test cell under the control condition with latent representation $z_{\mathrm{test}}^{\mathrm{ctrl}}$, we aim to predict its corresponding stimulated state by leveraging both the global transport plan and local similarity to the training control cells.

First, we construct a perturbation delta matrix $\Delta_{cs}\in R^{n\times d}$, where each row corresponds to the latent difference between a training control cell and its most relevant stimulated counterpart:

(15) $$\Delta_{cs}^{\left(i\right)}=\sum_{j=1}^{m}\gamma_{ij}\cdot \left(z_{j}^{\mathrm{stim}}-z_{i}^{\mathrm{ctrl}}\right)$$

This delta reflects how the *i*-th control training cell would respond under stimulation, as inferred by the transport plan γ.

Next, for a test control cell $z_{\mathrm{test}}^{\mathrm{ctrl}}$, we compute its similarity with all control cells in training set, resulting in a similarity vector $s\in R^{n}$. To determine the optimal similarity measure, we explored different alternatives, including cosine similarity, dot product, and Euclidean-based metrics. We finally determined cosine similarity as the most appropriate metric as it demonstrated consistently superior performance and is inherently scale-invariant. The predicted perturbation shift is then obtained by aggregating the training perturbation deltas weighted by these similarity scores:

(16) $$\Delta_{\mathrm{pred}}=\sum_{i=1}^{n}s_{i}\cdot \Delta_{cs}^{\left(i\right)}$$

where the similarity weights $\left\{s_{i}\right\}$ are normalized across the top-*k* most similar training cells.

The predicted latent representation in the stimulated state is computed by applying the aggregated delta:

(17) $${\hat{z}}_{\mathrm{pred},\mathrm{test}}^{\mathrm{stim}}=z_{\mathrm{test}}^{\mathrm{ctrl}}+\Delta_{\mathrm{pred}}$$

Finally, the predicted latent embedding is decoded into gene expression space using the decoder network:

(18) $${\hat{x}}_{\mathrm{pred},\mathrm{test}}^{\mathrm{stim}}=\mathrm{Decoder}\left({\hat{z}}_{\mathrm{pred},\mathrm{test}}^{\mathrm{stim}}\right)$$

## 3. Results

In this study, we employ multiple approaches to assess the performance of scOTM, primarily following the evaluation process from scGen [12] to ensure fairness. First, we utilize UMAP visualization to intuitively illustrate the similarity between predicted and actual stimulated cells [31], and then we conduct regression analysis to assess the agreement between predicted and actual [32]. Here, $R^{2}$ is computed as the squared Pearson correlation coefficient (*r*) between predicted and observed gene expression. We used the Fisher-*z* transformation [33] to estimate the 95% confidence intervals for *r* and then squared it to obtain the intervals for $R^{2}$, and the corresponding two-sided *p*-values were calculated for testing the null hypothesis r=0. These statistical measures are reported alongside the point estimates to provide a quantitative assessment of robustness. Additionally, we also evaluate the identification of differentially expressed genes (DEGs) to validate biological relevance. Finally, we assess the model’s explainability by clustering its latent representations, revealing cell condition states and biologically meaningful patterns captured by the model.

### 3.1. scOTM Accurately Predicts Perturbation Response Across Unseen Cell Types

**Figure 4:**
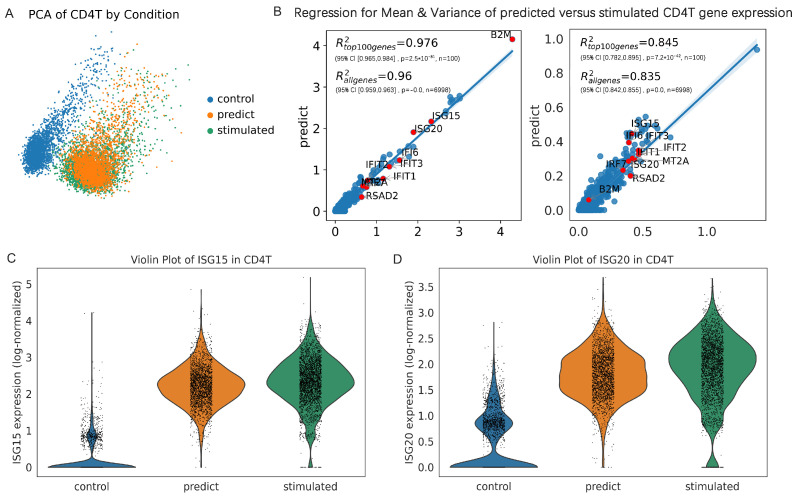
scOTM accurately predicts single-cell perturbation response. (**A**) UMAP visualization of CD4T cells under different conditions. (**B**) Linear regression between stimulated and predicted CD4T gene expression mean (left) and variance (right). For the mean expression regression, $R^{2}=0.961$ with 95% CI [0.959, 0.963], $p<1\times 10^{-300}$, across 6998 genes. For the variance regression, $R^{2}=0.849$ with 95% CI [0.842, 0.855], $p<1\times 10^{-300}$, across 6998 genes. (**C**) Violin plot of ISG15 top gene expression (log-normalized) distribution in control, predicted, and stimulated groups. (**D**) Violin plot of ISG20 top gene expression (log-normalized) distribution in control, predicted, and stimulated groups.

We selected CD4T as the representative cell type from the PBMC dataset [34] due to its interferon response and pivotal role in inflammation during lupus pathogenesis. To intuitively demonstrate the predictive performance of scOTM, we visualized its gene expression profiles using UMAP first. As depicted in Figure 4A, the predicted perturbed states generated by scOTM exhibit a strong overlap with the true stimulated states in the low-dimensional space, indicating accurate modeling of transcriptional changes under perturbation.

We next evaluated the predictive performance of scOTM through linear regression analyses comparing the mean and variance of gene expression between predicted and actual responses. Since the predicted and ground truth datasets are unpaired and lack one-to-one correspondence at the single-cell level, direct cell-wise comparison is not feasible. Therefore, we performed a gene-level regression analysis by comparing the mean expression of each gene between the predicted and actual perturbed responses, which allows us to evaluate how well each method captures global transcriptional shifts at the gene level. In addition to the mean expression, we also evaluated gene-wise variance to assess whether models captured not only central trends but also expression heterogeneity under perturbation (Figure 4B).

The results reveal a high consistency between the prediction and ground truth, with strong $R^{2}$ scores for the entire gene set ($R^{2}=0.976$). Importantly, the most prominent DEGs cluster tightly along the diagonal, indicating accurate modeling of condition-specific expression patterns. Furthermore, Figure 4C,D show violin plots of *ISG15* and *ISG20*, the top DEG, where the predicted distribution mirrors that of the stimulated group, confirming the high-fidelity of scOTM’s gene-level predictions.

### 3.2. scOTM Outperforms Alternative Approaches Across Unseen Cell Types

We evaluated the performance of scOTM in comparison with several state-of-the-art methods, including scPRAM [23], scGen [12], scVIDR [17], trVAE [16], and CellOT [21]. To ensure a fair and consistent comparison across methods, we standardized the training and testing datasets used in all evaluations. We adopted the default parameter settings provided by the publicly available implementations across all scenarios. In addition, due to incompatibilities between code versions and available implementations, several baseline methods could not be reliably reproduced. In such cases, we cite the original results reported in the scPRAM study to preserve the integrity and completeness of the comparison.

**Figure 5:**
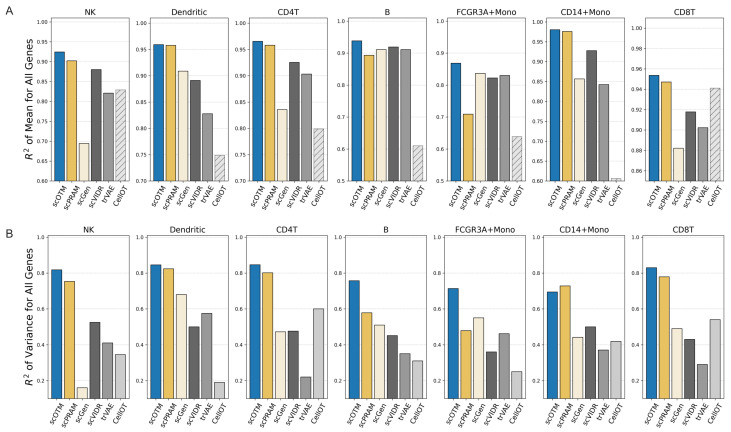
scOTM outperforms alternative methods across unseen cell types. (**A**) Bar plots comparing the mean $R^{2}$ values of gene expression across different cell types for all genes among various methods. All bars start from a baseline of 0, except for CellOT, which starts from 0.5 to visualize its absolute performance. The numeric values above CellOT bars represent their original $R^{2}$ scores. (**B**) Heatmaps displaying the variance $R^{2}$ values for all genes across different methods.

**Figure 6:**
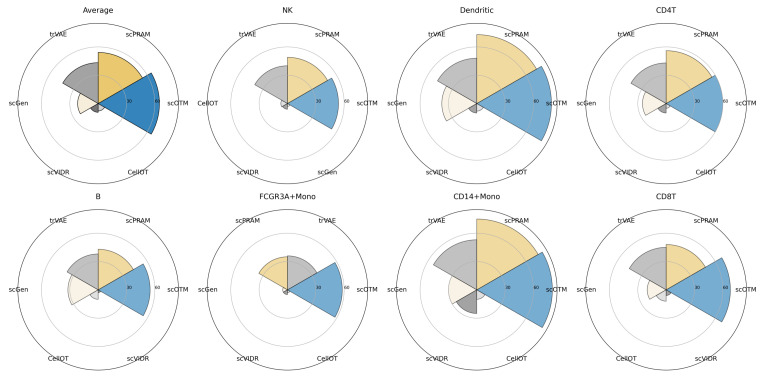
Comparison of the number of common DEGs identified by different methods across various immune cell types. The scOTM method consistently outperforms other methods in identifying DEGs with the top 100 ground-truth genes.

Figure 5 summarizes the performance of scOTM and other competing methods across seven cell types from the PBMC scRNA-seq dataset in out-of-sample prediction settings. Each bar represents the mean $R^{2}$ of all genes for a given cell type when it is held out during training and used for evaluation. The results showed that, in terms of the $R^{2}$ of regression for all gene mean expression, scOTM consistently achieved the highest predictive performance on 7/7 out-of-sample sub-experiments, with an overall average $R^{2}$ of 94.05%, ranging from 86.8% (for FCGR3A+ Mono) to 97.4%. And for the $R^{2}$ of variance regression, scOTM also gives better insights into the heterogeneity of single-cell gene expression compared to other methods, with an average $R^{2}$ of 77.5% (70.0–83.5%), achieving an average of 9.8% improvement compared to the second-best method scPRAM, with an average $R^{2}$ of 70.6% (47.9–82.3%). While scOTM shows marginally inferior performance compared to scPRAM in the CD14-positive monocyte (CD14+ Mono) cell type, it identifies a greater number of ground-truth DEGs (Figure 6).

**Figure 7:**
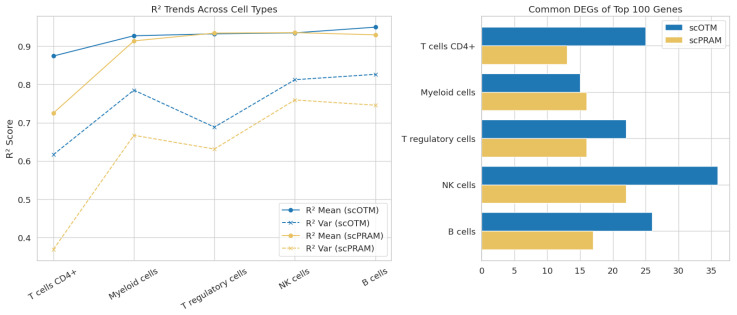
Performance comparison between scOTM and the second-best method, scPRAM, across all cell types in the Kaggle dataset. The left panel shows the R^2^; scores (mean and variance) of predicted gene expression across cell types, while the right panel shows the number of common DEGs identified among the top 100 genes. scOTM consistently outperforms scPRAM in both metrics across all cell types.

To further evaluate the performance of scOTM, we conducted a comprehensive evaluation on the Kaggle single-cell perturbation dataset. Specifically, we focused on cells treated with belinostat and their corresponding DMSO controls, resulting in a dataset comprising 8562 control and 8101 perturbed cells. We compared scOTM against scPRAM, the second-best method, across five major immune cell types: B cells, CD4+ T cells, NK cells, myeloid cells, and regulatory T cells. As shown in Figure 7, scOTM consistently outperformed scPRAM in predictive accuracy, achieving higher $R^{2}$ scores for both the mean and variance of gene expression across all cell types. In addition, scOTM recovered more common DEGs among the top 100, suggesting improved biological relevance of its predictions. These results demonstrate scOTM’s superior ability to capture transcriptional perturbation effects and generalize across heterogeneous cellular contexts.

### 3.3. scOTM Enhances the Accuracy of Differentially Expressed Gene Identification

To further assess the gene-level accuracy of scOTM, we compared the DEGs identified by each method against those derived from the observed stimulated data across all out-of-sample sub-experiments. The accurate recovery of DEGs is critical for downstream interpretation of perturbation-associated pathways. As shown in Figure 6, scOTM consistently identifies a substantially higher number of DEGs that overlap with the ground truth, outperforming all baseline methods across cell types. This advantage is particularly pronounced in dendritic cells and CD14+ monocytes, where scOTM successfully recovers 74 and 83 out of the top 100 DEGs, respectively.

**Figure 8:**
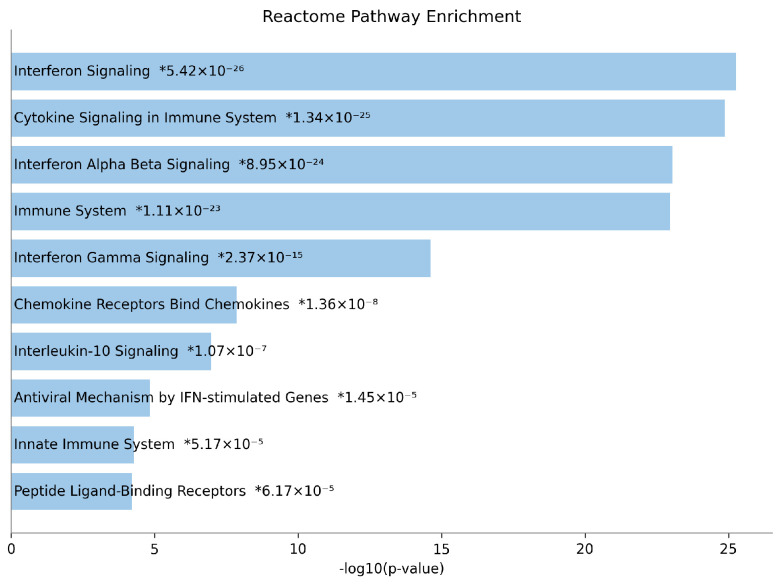
Gene enrichment analysis of the top 100 predicted DEGs by scOTM using the Reactome 2024 gene database.

We then performed pathway enrichment analysis using the Reactome 2024 gene library [35] (Figure 8) to investigate the biological relevance. The analysis was based on the top 100 DEGs identified by scOTM, with the x-axis of the enrichment plot representing −log_10_(*p*-value) and the y-axis denoting the significantly enriched pathways. The results highlight a strong enrichment of immune-related pathways, with prominent associations observed in interferon signaling and antiviral defense mechanisms. These findings are consistent with the IFN-β perturbation applied in the PBMC dataset, reinforcing the biological validity of scOTM’s predictions. Moreover, the presence of additional immune-regulatory pathways further substantiates the link between IFN-β stimulation and immune activation.

### 3.4. Interpretability of scOTM

**Figure 9:**
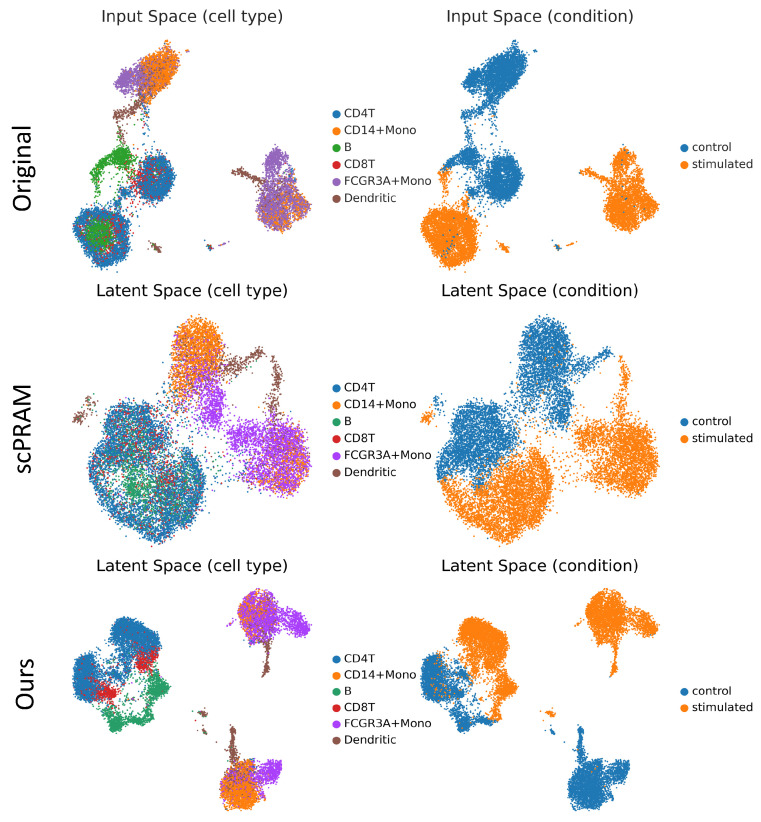
Comparison of data distributions and learned latent spaces, visualized by cell type (**left column**) and perturbation condition (**right column**). The top row shows the original input space, the middle row depicts the latent space learned by the second-best method scPRAM, and the bottom row shows the latent representation learned by our method.

In addition to its strong predictive performance, we also investigate the explainability of scOTM by visualizing the learned latent space representations. As shown in Figure 9, we compare the input space (top), the latent space learned by the second-best method scPRAM (middle), and the latent space learned by scOTM (bottom). Each representation is colored by either cell type (left column) or perturbation condition (right column) to reveal biological and experimental structure. In the input space, cells of different types and conditions exhibit partially overlapping distributions, with limited separation between control and stimulated states. While scPRAM improves the condition-based separation to a certain degree, it fails to clearly disentangle cell types, and there are overlapping and diffuse clusters in the latent space based on different conditions. In contrast, scOTM produces well-structured and interpretable latent representations. In the cell-type-colored latent space, each cell type forms a compact and distinct cluster, demonstrating that the model effectively preserves biological identity. In the condition-colored latent space, control and stimulated cells are clearly separated, revealing that scOTM captures a coherent and interpretable perturbation trajectory. The separation is sharper and more consistent across cell types compared to baseline methods. The compact cluster in the condition latent, combining the recovering shift strategy and OT matching, enables scOTM to generate more accurate perturbed results.

### 3.5. Sensitivity Analysis Under Varying Data Scales

To evaluate the robustness of scOTM, we performed a sensitivity analysis using datasets with varying scales [36]. Table 1 presents the results of this analysis, where we systematically subsampled the training data at five different proportions (10%, 30%, 50%, 70%, and 100%) and evaluated scOTM on two metrics: the mean $R^{2}$ of all genes and the number of common DEGs among the top 100 compared to the observed stimulated data.

As shown in Table 1 part (A), scOTM maintains strong predictive performance across all cell types, even when trained on as little as 10% of the data. The $R^{2}$ values generally increase as more training data are provided, indicating that scOTM benefits from larger datasets while already achieving high accuracy with limited samples. For instance, in the vast majority of cell types, the mean $R^{2}$ reaches 0.85 or higher, and often exceeds 0.94 even at moderate data scales. In Table 1 part (B), we observe a corresponding increase in the number of DEGs correctly recovered by scOTM. The DEG overlap rises consistently with data scale in nearly all cell types, confirming that a larger training set enhances gene-level resolution. For instance, scOTM identifies 74–82 of the top 100 DEGs in dendritic and CD14+ monocyte populations when trained on the full data, compared to 65–76 at 10%.

While the overall trend indicates that larger training sets lead to improved performance, we note that the FCGR3A+ Mono cell type exhibits decreases in $R^{2}$ and DEG overlap at higher data ratios. This phenomenon is primarily attributed to (i) inherent randomness in data sampling during subsetting, and (ii) an imbalanced distribution between conditions, where the number of stimulated cells greatly exceeds that of control cells (2501 vs. 1100), see Figure 2. To assess the frequency of this decrease, we repeated the analysis at these proportions with 10 independent random seeds and observed decreases in 3 out of 10 repeats. Nevertheless, the general upward trend across metrics and cell types supports the robustness and scalability of scOTM.

### 3.6. Ablation Analysis

To evaluate the impact of different modeling choices, we conducted two independent ablation experiments. The first focuses on the effect of MMD loss compared to KL divergence loss, while the second investigates the benefit of integrating LLM-derived single-cell profile embeddings. Each component is evaluated separately to understand its contribution to model performance across cell types and conditions. For the second ablation experiment involving LLM embeddings, we extended the number of training epochs to account for the increased input complexity and richer semantic information. Importantly, despite more training epochs, the only variable changed is still the presence or absence of the LLM embedding, ensuring a fair and controlled comparison.

#### 3.6.1. Ablation Results and Effectiveness of MMD Loss

To evaluate the impact of different distribution alignment strategies in the model, we conducted an ablation study comparing MMD loss with KL divergence loss. The results, as shown in Table 2, demonstrate that introducing the MMD loss consistently improves the predictive performance in most cell types. On average, the number of common DEGs in the top 100 increased from 60.43 to 64.71, indicating enhanced consistency in capturing cell-type-specific transcriptomic changes. More importantly, the mean $R^{2}$ of predicted gene expression (mean values) improved from 0.9145 to 0.9416, and the variance prediction improved from 0.7502 to 0.7682. These improvements highlight the effectiveness of MMD in aligning global feature distributions, as it compares full distributions (via kernel embeddings) rather than relying on parametric assumptions. While KL divergence focuses on local divergence between distributions and is sensitive to mismatched support, MMD provides a more stable and flexible regularization mechanism—especially beneficial in settings with complex perturbation responses.

#### 3.6.2. Ablation for Effectiveness of Combining LLM Embeddings

To assess the impact of incorporating LLM-derived perturbation embeddings, we conducted an ablation study across all immune cell types, as shown in Table 3. Overall, adding LLM embeddings improved model performance in both identifying the number of common DEGs among the top 100 and the predictive accuracy measured by the $R^{2}$ of the regression analysis of gene expression (mean and variance). Cell types such as FCGR3A+ Mono, B cells, and Dendritic cells demonstrated substantial gains in both expression mean and variance prediction, indicating that the semantic representation from pretrained language models effectively captured perturbation-related signals. The increase in common DEGs further suggests improved biological alignment in the predicted gene expression profiles. For CD14+ Mono and CD8T cells, however, the incorporation of LLM embeddings resulted in marginal or no improvement. This is likely due to the fact that the base model already achieved near-optimal performance on these cell types, with $R^{2}$ values exceeding 0.95 for expression means. In such cases, the predictive capacity is approaching the intrinsic performance ceiling imposed by the data itself, leaving little room for further gains.

These results confirm that integrating LLM-based perturbation representations enhances the model’s ability, particularly benefiting more heterogeneous or challenging cell populations.

### 3.7. Computational Resources and Runtime

All experiments were conducted on an NVIDIA GeForce RTX 3080 GPU with 10 GB of memory. GPU utilization and memory usage depend on the selected batch size; with the default batch size of 256, GPU utilization is approximately 40%, while with a minimum batch size of 64, utilization drops to around 10%. For runtime, processing datasets with about 20,000 cells and 7000 features typically takes 8–12 min per sub-experiment for one cell type, with slight variations depending on data size and model convergence speed. These computational requirements are modest and make the framework feasible for most modern desktop or workstation environments.

## 4. Conclusions and Discussion

In this work, we introduced scOTM, a generative modeling framework designed for predicting transcriptional responses to perturbations in single-cell data, especially under unpaired and unseen conditions. scOTM addresses the limitations of conventional variational autoencoders by using maximum mean discrepancy regularization instead of KL divergence constraints that impose a strict point-wise Gaussian prior, enabling the model to learn more flexible and biologically meaningful latent representations. scOTM employs optimal transport, allowing for a principled and interpretable alignment between control and perturbed cell populations, without relying on paired samples. In addition, a key advantage of scOTM lies in its integration of prior biological knowledge derived from large language models trained on molecular and single-cell corpora. This incorporation of external knowledge significantly enhances predictive performance, particularly in heterogeneous cell populations. Comprehensive evaluations and a series of ablation studies confirm the robustness and generalizability of our approach, demonstrating improvements in whole-transcriptome prediction, differentially expressed gene identification, and generalization across cell types.

Despite these advantages, a notable limitation of scOTM stems from its reliance on LLM-derived priors trained predominantly on human single-cell data, which may limit its applicability to non-human species. Nevertheless, ablation studies on the MMD module demonstrate that the model remains competitive even without LLM-based embeddings, indicating the robustness of scOTM’s core architecture. In addition, scaling scOTM to very large single-cell atlases (e.g., tens of millions of cells) may require substantial computational resources, including extended training time and high memory usage, which could necessitate distributed training strategies or model optimization for practical deployment. Overall, scOTM represents a flexible and interpretable framework that bridges generative modeling with domain-informed priors, offering a powerful tool for in silico perturbation analysis and advancing applications in drug discovery, disease modeling, and precision medicine. Looking forward, integrating batch-effect correction modules could further enhance cross-dataset generalization, while extending the framework to multimodal single-cell data (e.g., ATAC-seq, CITE-seq) holds promise for capturing a more comprehensive view of cellular states and regulatory mechanisms.

## Figures and Tables

**Table 1:** Sensitivity analysis of scOTM under varying data scales. Part (**A**) shows the mean $R^{2}$ values for all genes in each cell type. Part (**B**) reports the number of overlapping DEGs (out of the top 100) between the predicted and observed perturbed states.

Cell Type	0.1	0.3	0.5	0.7	1.0
(A) Mean $R^{2}$ of All Genes					
NK	0.8471	0.8947	0.9242	0.9111	0.9205
Dendritic	0.9025	0.9384	0.9493	0.9467	0.9638
CD4T	0.8782	0.9232	0.9421	0.9438	0.9608
B	0.8552	0.8925	0.9342	0.9372	0.9349
FCGR3A+ Mono	0.6311	0.7805	0.7641	0.6633	0.9292
CD14+ Mono	0.9451	0.9612	0.9676	0.9674	0.9678
CD8T	0.8913	0.9313	0.9443	0.9464	0.9546
(B) Common DEGs among Top 100 DEGs					
NK	47	50	54	56	56
Dendritic	65	74	73	75	78
CD4T	48	50	55	55	55
B	43	51	55	57	58
FCGR3A+ Mono	33	40	40	36	59
CD14+ Mono	76	80	82	81	81
CD8T	50	60	61	62	62

**Table 2:** Ablation study of MMD loss on single-cell perturbation prediction across cell types.

Cell Type	Common DEGs (Top 100)		$R^{2}$ of Regression Analyses	
	w/o MMD	with MMD	Expr. Mean	Expr. Variance
NK	52	54	0.8998 → 0.9225	0.7941 → 0.8172
Dendritic	71	79	0.9604 → 0.9648	0.8300 → 0.8042
CD4T	60	60	0.9606 → 0.9610	0.8488 → 0.8352
B	53	55	0.9246 → 0.9409	0.7174 → 0.7460
FCGR3A+ Mono	39	58	0.7259 → 0.8754	0.5271 → 0.7019
CD14+ Mono	80	81	0.9720 → 0.9743	0.6967 → 0.7027
CD8T	68	68	0.9537 → 0.9536	0.8072 → 0.8160
Mean	60.43	**65**	0.9141 → **0.9418**	0.7459 → **0.7748**

**Table 3:** Ablation study of incorporating LLM embeddings on single-cell perturbation prediction across cell types.

Cell Type	Common DEGs (Top 100)		$R^{2}$ of Regression Analyses	
	w/o LLM	with LLM	Expr. Mean	Expr. Variance
NK	54	55	0.9209 → 0.9244	0.8279 → 0.8377
Dendritic	75	77	0.9546 → 0.9608	0.7410 → 0.7485
CD4T	60	60	0.9554 → 0.9575	0.8223 → 0.8346
B	55	59	0.9230 → 0.9342	0.7097 → 0.7554
FCGR3A+ Mono	61	63	0.8940 → 0.9301	0.7080 → 0.7783
CD14+ Mono	83	81	0.9732 → 0.9661	0.6906 → 0.6916
CD8T	65	65	0.9533 → 0.9506	0.8259 → 0.8031
Mean	64.71	**65.71**	0.9392 → **0.9462**	0.7608 → **0.7785**

## Data Availability

All data are available online: the Kang PBMC dataset can be accessed from the Gene Expression Omnibus (GEO) database with identifier GSE96583 [26], and the Kaggle dataset can be downloaded from the Kaggle competition webpage (accessed on 17 August 2025) [27].
